# The ABCs of membrane transporters in health and disease (SLC series): Introduction^☆^^☆☆^

**DOI:** 10.1016/j.mam.2012.12.009

**Published:** 2013-04

**Authors:** Matthias A. Hediger, Benjamin Clémençon, Robert E. Burrier, Elspeth A. Bruford

**Affiliations:** a Institute of Biochemistry and Molecular Medicine, University of Bern, Bern, Switzerland; b Swiss National Centre of Competence in Research, NCCR TransCure, University of Bern, Bern, Switzerland; c Stemina Biomarker Discovery, Madison, WI, USA; d HUGO Gene Nomenclature Committee, EMBL-EBI, Wellcome Trust Genome Campus, Hinxton, Cambridgeshire CB10 1SD, United Kingdom

**Keywords:** Transporter, Carrier, Nomenclature, Solute carrier genes, SLC, Exchanger, Cotransporter, Uniporter, Symporter, Antiporter, Ion transport, Solute transport, Coupled transport, Channel, Pump, ABC transporter, Aquaporin, Water soluble vitamins, Structure, Membrane proteins, Glucose transporter, Diabetes, GLUT1, SLC2A1

## Abstract

The field of transport biology has steadily grown over the past decade and is now recognized as playing an important role in manifestation and treatment of disease. The SLC (solute carrier) gene series has grown to now include 52 families and 395 transporter genes in the human genome. A list of these genes can be found at the HUGO Gene Nomenclature Committee (HGNC) website (see www.genenames.org/genefamilies/SLC). This special issue features mini-reviews for each of these SLC families written by the experts in each field. The existing online resource for solute carriers, the Bioparadigms SLC Tables (www.bioparadigms.org), has been updated and significantly extended with additional information and cross-links to other relevant databases, and the nomenclature used in this database has been validated and approved by the HGNC. In addition, the Bioparadigms SLC Tables functionality has been improved to allow easier access by the scientific community. This introduction includes: an overview of all known SLC and “non-SLC” transporter genes; a list of transporters of water soluble vitamins; a summary of recent progress in the structure determination of transporters (including GLUT1/SLC2A1); roles of transporters in human diseases and roles in drug approval and pharmaceutical perspectives.

## 1 Overview membrane transporter genes

**Fig. 1:**
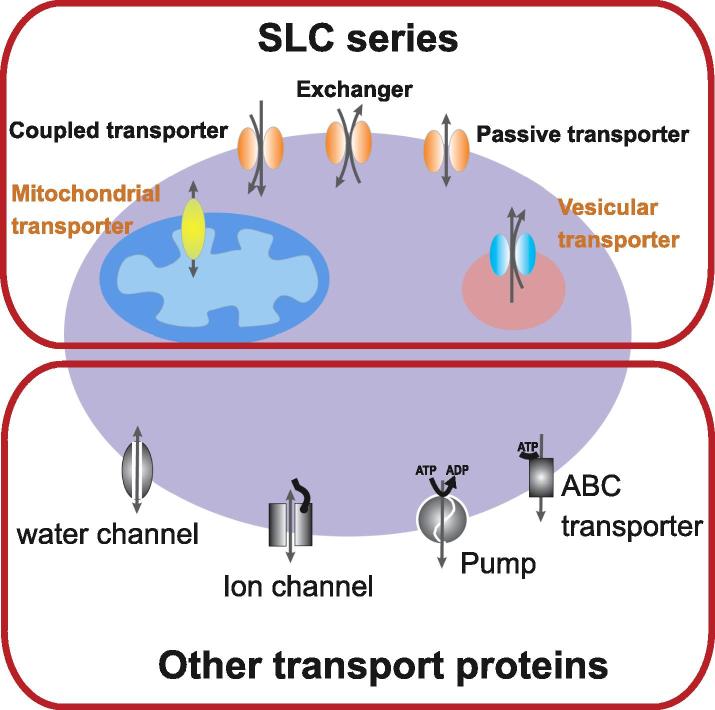
Cartoon showing a cell with SLC and non-SLC transporters expressed in the plasma membrane or in intracellular compartments. Note that the non-SLC transporters are also expressed in intracellular compartments.

The uptake and efflux by cells and organelles of crucial compounds such as sugars, amino acids, nucleotides, inorganic ions and drugs is controlled by transporters. Transporters have been called the gatekeepers of small molecules and can be divided based on passive or active mechanisms of function (Fig. 1). Passive transporters, also known as facilitated transporters, allow diffusion of solutes (e.g., glucose, amino acids, urea) across membranes down their electrochemical gradient. Active transporters create solute gradients across membranes, utilizing diverse energy-coupling mechanisms. These active transporters are classified as primary- or secondary-active transporters according to the directness of coupling to cellular energy (e.g., ATP hydrolysis).

Primary-active, ATP-dependent transporters include members of the ABC (ATP-binding cassette) transporter family and ion pumps (ATPases). Mammalian ABC transporters [e.g., P-glycoprotein/MDR (multi-drug resistance) proteins, TAP (transporter associated with antigen processing)] bind and hydrolyze ATP for the transport of a variety of substances such as ions, carbohydrates, lipids, xenobiotics and drugs out of cells or into cellular organelles (Borst and Elferink, 2002). Ion pumps hydrolyze ATP to actively pump ions such as Na^+^, K^+^, H^+^, Ca^2+^ and Cu^2+^ out of cells or into organelles (Cox and Moore, 2002, Dunbar and Caplan, 2000, Muller and Gruber, 2003). These pumps also generate and maintain electrochemical ion gradients across membranes. Such ion gradients are in turn used by “secondary-active” ion-coupled transporters to drive uphill transport of nutrients across biological membranes.

Similar to transporters, channels allow movement of solutes down their electrochemical gradients (Armstrong, 2003, Chen, 2003, Decoursey, 2003, Gunther et al., 2003, Jiang et al., 2003, Peng et al., 2003, Yu and Catterall, 2003). Transporters typically have a fixed stoichiometry of ion(s)/solute(s) movement per translocation cycle. Ion or solute flow through channels, on the other hand, is controlled by the open probability of the channel via gating mechanisms and the single channel conductance (number of charges per second at a given voltage).

In the past, the molecular identification of transporters has lagged compared to other protein classes because of their membrane localization, hydrophobic nature, and relatively low abundance, although physiological data for a variety of transporters have been reported extensively. It has now been over two decades since the expression cloning approach for transporters was developed (Hediger et al., 1987, Romero et al., 1998), when the transporter field underwent a phase of rapid gene identification using this and subsequent homology approaches. The growth in genes encoding solute carriers (SLC) that have been identified has led to a deeper understanding of how they have evolved from common ancestral genes and now form families that can be grouped and classified. In fact there are many genes that have been identified and fall within the existing classified families based on sequence similarities, but their ligands and metabolic functions still remain obscure.

### 1.1 The SLC gene nomenclature system

The SLC gene nomenclature system was originally established in the 1990s by Matthias Hediger in collaboration with Phyllis McAlpine, the first chair of the HGNC, and has since been maintained and extended in collaboration with Elspeth Bruford who now leads the HGNC. In 2004, a special mini-review series was commissioned to provide an overview of the different types of mammalian transport systems belonging to the SLC series (Hediger et al., 2004). Since that initial publication, there has been increased interest in the SLC genes as their importance in both health and disease has become apparent. Therefore, it is completely appropriate that, after 8 years of solid progress in this field, the entire series is reviewed again. Additional knowledge in the areas of gene expression, regulation, protein structure, function, and roles in various disease states are the focuses for the reviews in this series. In addition, due to the role of many SLC family members in drug disposition, transporters are now routinely being evaluated as part of the drug development and approval process, and are receiving attention from regulatory authorities around the world.

### 1.2 SLC and “non-SLC” transporter genes

The list of currently approved SLC human gene families is shown in Table 1. In comparison to the known members in 2004, the number of recognized gene families has grown from 43 to 52 and the total number of human genes now stands at 395, an increase of nearly 100 genes. In general, the genes are named using the root symbol SLC, followed by a numeral (e.g., SLC1, solute carrier family 1), followed by a letter which defines the subfamily (only A is used when the family has not been subdivided) and finally a number designating the individual transporter gene (e.g., *SLC3A1*). Transporters are assigned to a specific family if the encoded protein has at least 20% amino acid sequence identity to other members of that family. A few exceptions to the classifications above exist within the SLC series, including the SLC51 family which consists of two members, *SLC51A* and *SLC51B*, that are not related by sequence similarity but instead encode the two subunits (alpha and beta) of the organic solute transporter. Another naming deviation is the SLC21 organic anion transporter family which now uses the root symbol SLCO, an update made to accommodate a unique species-independent classification system that is necessary because this family has been the subject of rapid evolution (Hagenbuch and Stieger, 2013). Indeed, while the SLC series was originally developed for human genes, some other SLC families have been expanded to include genes that are only found in rodents (e.g. *Slc7a12* and multiple members of the SLC22 family) or even in insects (*slc18a4* has been identified in Drosophila and honey bee). While orthologs of human genes commonly use the same designation as the human gene, though the case of the symbol may vary (e.g., the rodent ortholog of human *SLC2A1* is denoted as *Slc2a1*), we anticipate that as more animal genomes are sequenced and annotated, the SLC series will expand appropriately to encompass novel genes that are not orthologs of human solute carriers.

**Table 1:** List of currently approved SLC families. The total numbers of members in each family are shown on the right. For detailed information about the SLC genes, please visit: http://www.bioparadigms.org.

The HGNC Solute Carrier Family Series	Total 2004	Total 2013
SLC1: The high affinity glutamate and neutral amino acid transporter family	7	7
SLC2: The facilitative GLUT transporter family	14	14
SLC3: The heavy subunits of the heteromeric amino acid transporters	2	2
SLC4: The bicarbonate transporter family	10	10
SLC5: The sodium glucose cotransporter family	8	12
SLC6: The sodium- and chloride-dependent neurotransmitter transporter family	16	21
SLC7: The cationic amino acid transporter/glycoprotein-associated amino-acid transporter family	14	14
SLC8: The Na^+^/Ca^2+^ exchanger family	3	3
SLC9: The Na^+^/ H^+^ exchanger family	8	13
SLC10: The sodium bile salt cotransport family	6	7
SLC11: The proton coupled metal ion transporter family	2	2
SLC12: The electroneutral cation-Cl cotransporter family	9	9
SLC13: The human Na^+^-sulfate/carboxylate cotransporter family	5	5
SLC14: The urea transporter family	2	2
SLC15: The proton oligopeptide cotransporter family	4	5
SLC16: The monocarboxylate transporter family	14	14
SLC17: The vesicular glutamate transporter family	8	9
SLC18: The vesicular amine transporter family	3	4
SLC19: The folate/thiamine transporter family	3	3
SLC20: The type III Na^+^-phosphate cotransporter family	2	2
SLC21/SLCO: The organic anion transporting family	11	12
SLC22: The organic cation/anion/zwitterion transporter family	18	23
SLC23: The Na^+^-dependent ascorbic acid transporter family	4	4
SLC24: The Na^+^/(Ca^2+^–K^+^) exchanger family	5	6
SLC25: The mitochondrial carrier family	27	53
SLC26: The multifunctional anion exchanger family	10	11
SLC27: The fatty acid transport protein family	6	6
SLC28: The Na^+^-coupled nucleoside transport family	3	3
SLC29: The facilitative nucleoside transporter family	4	4
SLC30: The zinc efflux family	9	10
SLC31: The copper transporter family	2	2
SLC32: The vesicular inhibitory amino acid transporter family	1	1
SLC33: The Acetyl-CoA transporter family	1	1
SLC34: The type II Na^+^-phosphate cotransporter family	3	3
SLC35: The nucleoside-sugar transporter family	17	30
SLC36: The proton-coupled amino acid transporter family	4	4
SLC37: The sugar-phosphate/phosphate exchanger family	4	4
SLC38: The System A & N, sodium-coupled neutral amino acid transporter family	6	11
SLC39: The metal ion transporter family	14	14
SLC40: The basolateral iron transporter family	1	1
SLC41: The MgtE-like magnesium transporter family	3	3
SLC42: The Rh ammonium transporter family (pending)	3	3
SLC43: Na^+^-independent, system-L like amino acid transporter family	2	3
SLC44: Choline-like transporter family		5
SLC45: Putative sugar transporter family		4
SLC46: Folate transporter family		3
SLC47: Multidrug and Toxin Extrusion (MATE) family		2
SLC48: Heme transporter family		1
SLC49: FLVCR-related transporter family		4
SLC50: Sugar efflux transporters		1
SLC51: Transporters of steroid-derived molecules		2
SLC52: Riboflavin transporter family		3
Total	298	395

### 1.3 List of human transporter/channel genes

**Fig. 2:**
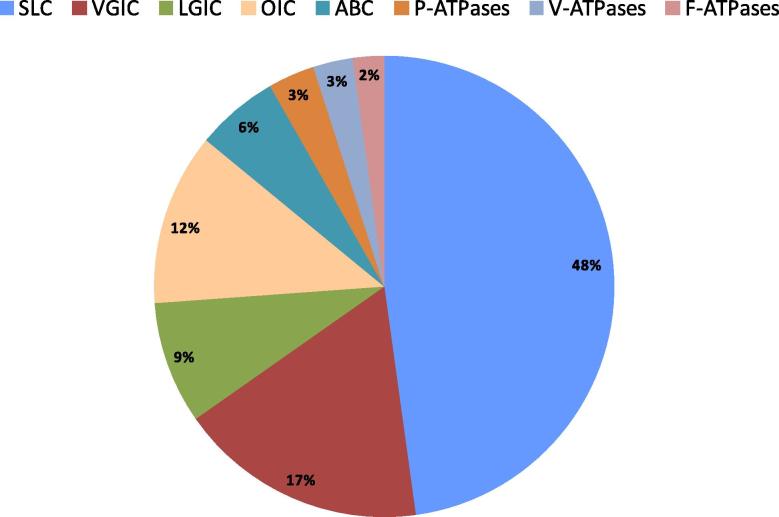
Pie chart depicting the proportion of genes encoding transporter-related proteins (total number: 826). SLC = solute carrier; VGIC = voltage gated ion channels; LGIC = ligand gated ion channels; OIC = other ion channels (e.g. aquaporins, connexins); ABC = ABC transporters; P-ATPases = P-type ATPases; V-ATPases = V-type ATPases; F-ATPases = F-type ATPases.

It is generally assumed that about 10% (∼2000) of all human genes are transporter-related, consistent with the biological significance of transporters and their roles in cell homeostasis. The SLC families represent a considerable portion of these genes: almost 400 different human genes fall into the solute carrier classification (Table 1, Table 2; and http://www.bioparadigms.org/slc/intro.htm) and additional SLC transporters are still being identified and their physiological roles elucidated. Besides transporter genes belonging to the SLC series, genes related to membrane transport include those encoding ion channels & ionotropic receptors, ABC transporters and ATP-driven transporters (Fig. 1, Fig. 2). As shown in Table 2, all genes from these categories taken together mount up to more than 800, close to 5% of the estimated complement of human protein coding genes. The remaining 5% of predicted transporter-related genes likely include other transporter – related genes as well as accessory gene products such as interacting partners, β subunits, regulatory proteins, etc.

**Table 2:** Current list of human transporter/channel genes (data from the HUGO Gene Nomenclature Committee, HGNC). From a total of 129 human ATPase genes, only 27 of the P-type and all 23 of the V-type are known to be transporting. 18 genes of the mitochondrial F1F0 proton ATPase contribute to the formation of a single pump.

Total number of human protein coding genes in HGNC dataset:	19047
	
Total number of human protein coding SLC genes	395
Total number of human protein coding ion channels & ionotropic receptor genes	315
Total number of human protein coding ABC genes	48
Total number of human protein coding transport-related ATPases	68
**Total**	**826**

Breakdown of “ion channels & ionotropic receptor genes”:	
Voltage gated ion channels (including 28 TRPs and 13 sodium channels)	144
Ligand-gated ion channels	71
Other ion channels (incl. 14 aquaporins, 22 connexins, 9 CLC chloride channels)	100
**Total**	**315**

Breakdown of “transport-related ATPases”:	
P-type	27
V-type	23
F-type	18
**Total**	**68**

## 2 Transporters of water soluble vitamins

Over the past several years, significant progress has been achieved identifying nutrient transporters such as those of trace minerals, vitamins, sugars, amino acids, etc. In particular, our understanding of the transport of water soluble vitamins has been greatly improved in recent years, as summarized in Table 3. In fact, numerous proteins implicated in their intestinal absorption as well as transport into cells of systemic tissues belong to SLC families and function as proton or sodium cotransporters. In some cases, ABC transporters (e.g. ABCC1 or ABCC3) present in the basolateral membrane of intestinal epithelial cells mediate the transport of some vitamins into the blood. Of special note is the transport mechanism of cobalamin (vitamin B12) that is mainly based on receptor-mediated endocytosis at the brush border membrane. With respect to the transport of vitamin B6, although the expression of the yeast *(S. pompe*) gene *bsu1*^+^ has been associated with pyridoxine (vitamin B6) uptake in yeast cells and the encoded protein (bsu1, XP_001713084.1) works as a proton cotransporter, its human homologue remains to be elucidated (Stolz et al., 2005). A sequence alignment analysis with human proteins reveals that bsu1 is most homologous to members of the human SLC22 family of organic cation/anion/zwitterion transporters. Specifically, yeast bsu1 shares 25% amino acid identity and 40% homology with human SLC22A15 (FLIPT1), with a sequence coverage of 32%.

**Table 3:** List of transporters known to be involved in the transport of water soluble vitamins. Most proteins implicated in their intestinal absorption and transport into cells of systemic tissues in fact belong to SLC families and function as proton or sodium cotransporters.

	Transporter				References	
	Intestine		Systemic tissues		Molecular Aspects of Medicine SLC mini-review series, 2013	Other
Vitamin	Apical	Basolateral				
Thiamine (B1)	SLC19A3 (ThTr2), facilitated transporter	SLC19A2 (ThTr1), facilitated transporter	SLC19A2 (ThTr1), facilitated transporter	Ubiquitous expression	Zhao and Goldman (2013)	Boulware et al., 2003, Dutta et al., 1999, Neufeld et al., 2001, Reidling et al., 2010, Said et al., 2004
Riboflavin (B2)	SLC52A3 (RFVT3), cotransporter (proton)	SLC52A1 (RFVT1), unknown	SLC52A2 (RFVT2),unknown	Brain > salivary gland > several tissues	Yonezawa and Inui (2013)	Foraker et al., 2003, Subramanian et al., 2011a, Subramanian et al., 2011b, Yamamoto et al., 2009, Yao et al., 2010
		SLC52A2 (RFVT2),unknown				

Niacin (B3)	SLC5A8 (SMCT1), cotransporter (sodium), differential expression unknown		SLC5A8 (SMCT1), cotransporter (sodium)	Kidney, brain, retina, muscle	Wright (2013)	Coady et al., 2007, Miyauchi et al., 2004
Pantothenic acid (B5)	SLC5A6 (SMVT), cotransporter (sodium),differential expression unknown		SLC5A6 (SMVT), cotransporter (sodium)	Brain, heart, kidney, lung, placenta	Wright (2013)	de Carvalho and Quick, 2011, Wang et al., 1999
Pyridoxine (B6)	Unknown		Unknown		−	Stolz et al. (2005)

Biotin (B7)	SLC5A6 (SMVT), cotransporter (sodium),differential expression unknown		SLC5A6 (SMVT), cotransporter (sodium)	Brain, heart, kidney, lung, placenta	Wright (2013)	de Carvalho and Quick, 2011, Wang et al., 1999

Folate (B9)	SLC46A1 (PCFT), cotransporter (proton)	ABCC3 (MRP3), ABC transporter	SLC19A1 (RFC), exchanger (organic phosphates)	Ubiquitous expression	Zhao and Goldman (2013)	(Kitamura et al., 2010, Matherly and Goldman, 2003, Matherly et al., 2007, Zhao et al., 2011, Zhao et al., 2009
			FOLR1 (FRα), receptor-mediated endocytosis	Kidney, choroid plexus, retina, brain, placenta		(Birn et al., 2005, Chancy et al., 2000, Kamen and Smith, 2004, Spiegelstein et al., 2004, Zhao et al., 2011, Zhao and Goldman, 2003
			FOLR2 (FRβ), receptor-mediated endocytosis	Kidney, choroid plexus, retina, brain, placenta, liver		(Elnakat and Ratnam, 2004, Paulos et al., 2004, Piedrahita et al., 1999, Ross et al., 1999, Wang et al., 2000a

Cobalamin (B12)	Cubam receptor complex, receptor-mediated endocytosis	ABCC1 (MRP1), ABC transporter	Transcobalamin receptor, receptor-mediated endocytosis	Ubiquitous expression	−	Beedholm-Ebsen et al., 2010, Fyfe et al., 2004, Moestrup et al., 1998, Quadros et al., 2009
Ascorbic acid (C)	SLC23A1 (SVCT1),cotransporter (sodium)	Unknown	SLC23A1 (SVCT1),cotransporter (sodium)	Epithelial tissues including kidney, liver, lung, skin	Bürzle et al. (2013)	Biondi et al., 2007, Corpe et al., 2005, Lee et al., 2006, Luo et al., 2008, Mackenzie et al., 2008, Tsukaguchi et al., 1999, Wang et al., 2000b, Wilson, 2005
			SLC23A2 (SVCT2),cotransporter (sodium)	Widespread, including brain, retina, placenta, spleen, prostate, testis, ovary		Biondi et al., 2007, Clark et al., 2002, Corpe et al., 2005, Godoy et al., 2007, Luo et al., 2008, Rajan et al., 1999, Tsukaguchi et al., 1999, Wilson, 2005

## 3 Progress in the structural biology of transporter proteins

**Fig. 3:**
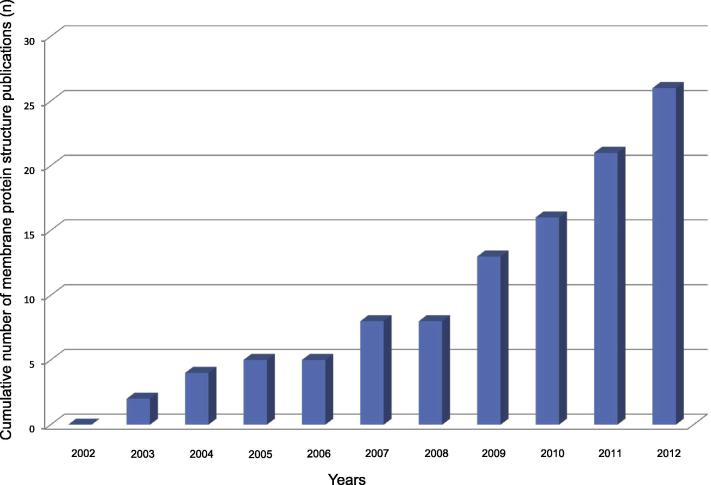
SLC family member crystal structures published since 2002. The histogram depicts the exponential evolution of the number of SLC family member crystal structures published in the last 10 years. The graph is based on information obtained from this SLC mini-review series.

Although all membrane proteins together represent about one third of the whole proteome and two thirds of all current therapeutic targets, the number of elucidated membrane protein structures is exceedingly small with about 280 unique structures deposited to date in the ‘Protein Data Bank’ (PDB) (available from www.rcsb.org/), representing less than 1% of all available 3D-resolved structures. The under-representation comes from the hydrophobic segments of integral membrane proteins that are embedded in phospholipid bilayers, making membrane proteins difficult to express and crystallize. Due to this issue, the structure determination of membrane proteins has long been considered to be a very difficult or even impossible task. However, recently developed new strategies and techniques have helped to improve the situation. Indeed, over the past decade, a large and increasing number of high-resolution structures have been solved, including numerous homologues of SLC family members (Fig. 3).

Those crystal structures, mainly obtained from lower organisms such as bacteria due to issues of expression, purification, and crystallization, can be used to model the putative structures of human homologues. Such analysis can be used to facilitate targeted mutagenesis approaches used to identify functional domains and substrate/ligand binding sites. An example is the modeling of the structure of human GLUT1 (SLC2A1) from its bacterial homologue (XylE) described below. From a pharmaceutical point of view, knowledge of the 3D-structures of human membrane proteins provides the starting point for structure-based drug design. This computational approach defines the topographies of complementary surfaces to which ligands bind. The obtained information can be used to assist development of novel compounds with improved potency and selectivity. This is nicely illustrated by the recent structure of a bacterial SLC1 homologue bound by the inhibitor DL-TBOA (SLC1 mini-review (Kanai et al., 2013)). Finally, knowledge of the structures of membrane proteins also opens the door to a better understanding of their functional mechanisms, as exemplified in the case of the SLC14 homologue dvUT (Shayakul et al., 2013). All of the recently discovered high resolution structures represent milestones in membrane transporter research and are described in detail in the following SLC mini-review series. A short overview is given in the Table 4.

**Table 4:** Crystal structures of SLC family members. This table gives an overview of available SLC family member structures based on information obtained from this mini-review series. This list describes general structural data with the name of the crystallized homologues. The origins of the homologues and the expression hosts are also mentioned. Information about the oligomeric state of the protein in the crystal, its substrate, if co-crystalized, its resolution (Å) and protein data bank accession number are also provided.

**Crystal structures of SLC family members**							
Human SLC family name	Homologue name	Homologue/expression system	Oligomeric state	Co-crystallization	Resolution	Protein Data Bank (PDB) accession#	References
SLC1	Glt_Ph_	*Pyrococcus horikoshii*/Bacteria	Trimer	l-glutamate	3.5 Å	1XFH	Yernool et al. (2004)
				l-aspartate, Sodium	3.29 Å	2NWX	Boudker et al. (2007)
				DL-Threo-Beta-Benzyloxyaspartate (DL-TBOA)	3.2 Å	2NWW	
				l-aspartate	2.96 Å	2NML	

SLC2	XylE	*Escherichia coli*/Bacteria	–	d-xylose	2.8 Å	4GBY	Sun et al. (2012)
				d-glucose	2.9 Å	4GBZ	
				6-bromo-6-deoxy-D-glucose	2.6 Å	4GCO	

SLC3/7	AdiC	*Salmonella enterica subsp.*/Bacteria	Tetramer	FAB fragment	3.2 Å	3NCY	Fang et al. (2009)
		*Escherichia coli*/Bacteria	Dimer	–	4 Å	3LRC	Gao et al. (2009)
				l-arginine	3 Å	3L1L	Gao et al. (2010)
				–	3 Å	3OB6	Kowalczyk et al. (2011)
	ApcT	*Methanocaldococcus jannaschii*/Bacteria	–	–	2.32 Å	3GIA	Shaffer et al. (2009)
				FAB fragment	2.48 Å	3GI9	
					2.59 Å	3GI8	
	GadC	*Escherichia coli*/Bacteria	Dimer	–	3.1 Å	4DJK	Ma et al. (2012)

SLC6	LeuT	*Eubacterium Aquifex aeolicus*/Bacteria	–	Tricyclic antidepressant (TCA) clomipramine	1.85 Å	2Q6H	Singh et al. (2007)
				l-leucine, Sodium, Imipramine	1.7 Å	2Q72	
	Mhp1	*Microbacterium liquefaciens*/Bacteria	–	––	3.8 Å	2X79	Shimamura et al. (2010)

SLC8/24	NCX_MJ	*Methanococcus jannaschii*/Bacteria	–	–	1.9 Å	3V5U	Liao et al. (2012)
SLC9	NhaA	*Escherichia coli*/Bacteria	–	–	3.45 Å	1ZCD	Hunte et al. (2005)
SLC10	ASBT_NM_	*Neisseria memingitidis*/Bacteria	–	Taurocholate	2.2 Å	3ZUX	Hu et al. (2011)
SLC13	VcINDY	*Vibrio cholerae*/Bacteria	Dimer	Citrate	3.2 Å	4F35	Mancusso et al. (2012)
SLC14	dvUT	*Desulfovibrio vulgaris*/Bacteria	–	*N,N’*-dimethylurea	2.4 Å	3K3F	Levin et al. (2009)
SLC15	PepT_So_	*Shewanella oneidensis*/Bacteria	–	–	3.6 Å	2XUT	Newstead et al. (2011)
SLC16	GlpT	*Escherichia coli*/Bacteria	–	–	3.3 Å	1PW4	Huang et al. (2003)
SLC23	UraA	*Escherichia coli*/Bacteria	–	Uracil	2.8 Å	3QE7	Lu et al. (2011)

SLC25	ANT1	*Bos taurus*/−	–	Carboxyatractyloside (CATR)	2.2 Å	1OKC	Pebay-Peyroula et al. (2003)
	UCP2⁎	*Mus musculus*/Bacteria	–	GDP	–	2LCK	Berardi et al. (2011)

SLC28	vcCNT	*Vibrio cholerae*/Bacteria	Trimer	Uridine	2.4 Å	3TIJ	Johnson et al. (2012)
SLC30	YiiP	*Escherichia coli*/Bacteria	Dimer	Zinc	3.8 Å	2QFI	Lu et al. (2009)

SLC42	AmtB	*Escherichia coli*/Bacteria	Trimer	–	1.4 Å	1U7G	Khademi et al. (2004)
	Rh	*Nitrosomonas europaea*/Bacteria	Trimer	Carbon dioxide	1.85 Å	3B9Z	Li et al. (2007)

SLC47	NorM	*Vibrio cholerae*/Bacteria	–	–	3.65 Å	3MKT	He et al. (2010)

⁎ Structure determined by NMR methods.

**Fig. 4:**
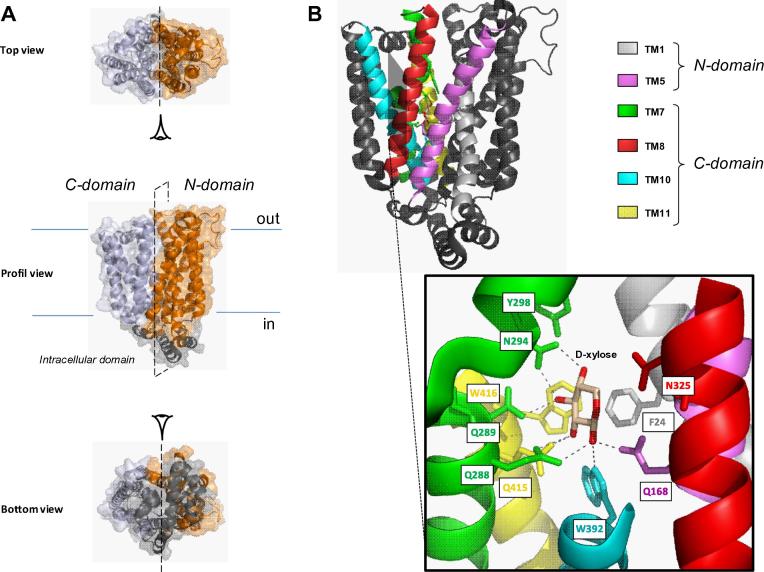
Crystal structure of XylE bound to d-xylose. (A) Three different views of cartoon representations and surface modeling of XylE in complex with d-xylose (PDB ID: 4GBY) by PyMOL v0.99 software. The structure of this bacterial homologue is divided into two distinct protomers (N- and C-domain) colored in orange and silver, respectively. Both domains are connected by an intracellular domain represented in gray. (B) Cartoon representation of XylE bound to d-xylose. Important transmembrane segments (TMs) involved in the binding site are colored (above, see legends). The binding site is formed by amino acids F24 (TM1), Q168 (TM5), Q288/Q289/N294/Y298 (TM7), N325 (TM8), W392 (TM10) and Q415/W416 (TM11), represented as sticks. The hydrogen bonds are depicted as dotted gray lines (below).

Each SLC mini-review includes a section describing structural aspects of its family members. A very recent publication on counterpart structures of the SLC2 family could not be added in this SLC mini-review series and therefore should be mentioned here. The high resolution structures of an *Escherichia coli* homologue (XylE) of human GLUT1-4 (SLC2A1-4) that shares about 30% sequence identity and 50% similarity was obtain in complex with d-xylose (PBD ID: 4GBY), d-glucose (PDB ID: 4GBZ) and 6-bromo-6-dexoxy-d-glucose (PDB ID: 4GCO) by X-ray crystallography methods (Sun et al., 2012). XylE is a proton-coupled d-xylose symporter belonging to the major facilitator superfamily (MFS). It is composed of 12 transmembrane segments (TMs) separated into two distinct protomers (N- and C-domain) that are connected by an intracellular domain comprising four helices (Fig. 4A). TM7 and TM10 are characterized by the particularity to represent discontinuous helices. This suggests that they provide the protein with the required flexibility for functional transport. The structure of XylE co-crystalized with its substrate d-xylose was determined at a resolution of 2.8 Å. The binding site was localized in the center of the TMs where d-xylose interacts mainly with the C-domain protomer mediated by TMs 7, 8, 10 and 11 (Fig. 4B, upper part). d-xylose is coordinated by polar residues interacting with the hydroxyl groups through eight hydrogen bonds corresponding to Q168 (TM5), Q288/Q289/N294 (TM7), W392 (TM10) and Q415 (TM11) (Fig. 4B, lower part). The aromatic residues F24 (TM1), Y298 (TM7), W392 (TM10) and W416 (TM11) are involved in the stabilization of the substrate. N325 (TM8) has also shown to be part of the binding site. Two other crystal structures of XylE bound to d-glucose and its derivative 6-bromo-6-dexoxy-d-glucose (6-BrGlc) were obtained at resolutions of 2.9 and 2.6 Å, respectively. However, d-glucose was not transported and was shown to inhibit d-xylose uptake. Interestingly, d-glucose bound around the same position as d-xylose. All amino acids comprising the d-xylose binding site are conserved with the exception of N325 (TM8) and involved new residues such as I171/Q175 (TM5) and F383/G388 (TM10).

The structure of XylE permitted the modelling of a predictive structure of human GLUT1 (SLC2A1) which differs drastically from previous models. Sequence alignment analysis shows that all amino acids are conserved in human GLUT1 except Q175 which is replaced by I168. The corresponding amino acids are listed in Table 5. This discovery raises new questions regarding the real identity of the d-glucose binding site in the human homologues.

**Table 5:** Conserved amino acids involved in the d-glucose binding site. The table shows that most amino acids involved in the d-glucose binding site in the bacterial homologue (XylE) are conserved in human GLUT1. Nevertheless, Q175 is not conserved in the human homologue and is represented by a dash (–). An asterisk (*) indicates homology.

	TM1	TM5	TM7	TM10	TM11
XylE	F24	Q168/I171/Q175	Q288/Q289/N294/Y298	F383/G388/W392	Q415/W416
GLUT1	F26	Q161/I164/-	Q282/Q283/N288/Y292	F379/G384/W388	N411^∗^/W412

## 4 Transporters and diseases

Given the increased understanding of the roles of transporters in normal physiology and disease, a focus of many of the mini-reviews in this series is to elaborate the latest information in this area. In addition to the following mini-reviews, an up to date summary of the knowledge can also be found by visiting the Bioparadigms website (www.bioparadigms.org). What follows is a summary of recent examples of pathologies associated with genetic defects of SLC genes:

### 4.1 Glutamate transport (SLC1 family)

As stated in detail in the corresponding mini-review by Kanai et al. (Kanai et al., 2013), glutamate transporters belonging to the SLC1 family play a critical role in the central nervous system by maintaining extracellular glutamate concentrations below excitotoxic levels and therefore may represent important drug targets. From this family, *SLC1A2* (GLT1) is involved in the pathogenesis of amyotrophic lateral sclerosis (ALS) as well as Alzheimer disease (AD). Large-scale genetic analyses for disease-associated genes have indicated a link between the *SLC1A2* (GLT1) gene and autism. The SLC1 family member *SLC1A3* (GLAST) has been linked to the pathogenesis of schizophrenia. Under pathological conditions such as ischemia after a stroke, the neuronal glutamate transporter SLC1A1 (EAAC1) is likely to run in reverse. Therefore, blockage of “reversal glutamate transport” by EAAC1 under ischemic conditions, using a glutamate transporter subtype-specific inhibitor may lead to a possible therapeutic strategy to prevent excitotoxicity under ischemic conditions.

### 4.2 Urate transport (SLC2 family)

Mueckler and Thorens explain in their SLC2 mini-review (Mueckler and Thorens, 2013) that although initially considered a glucose or fructose transporter, SLC2A9 (GLUT9) is now established as a urate transporter. Uric acid levels have been associated with metabolic syndrome and early-onset nephropathy and, indeed, genetic defects of *SLC2A9* are linked to these pathologies.

### 4.3 Neurotransmitter transport (SLC6 family)

In their mini-review on SLC6 transporters, which transport substrates such as serotonin, dopamine, norepinephrine, GABA, taurine, creatine, as well as amino acids, Pramod et al. (Pramod et al., 2013) discuss epigenetics research as a possible way to understand diseases that do not have a clear genetic etiology. Given the relationship of SLC6 transporters to mood disorders such as depression, addiction, aggression, post-traumatic stress disorder (PTSD), anxiety, obsessive compulsive disorder (OCD), and disorders such as attention deficit hyperactivity disorder (ADHD) and autism, the possibility that these illnesses may be beyond simple genetic control where environmental imprinting on gene expression could modulate the severity or propensity for developing these conditions suggests epigenetics could provide an exciting new option for therapeutic intervention.

### 4.4 Di- and tri-carboxylate/sulfate transport (SLC13 family)

As summarized in the SLC13 family mini-review about Na^+^-coupled di- and tri-carboxylate/sulfate transporters by Bergeron et al. (Bergeron et al., 2013), major (patho)physiological roles are attributed to *SLC13A2* (NaDC1), including renal handling of citrate and nephrolithiasis. In addition *SLC13A3* (NaDC3) has been suggested to participate in the pathogenesis of the two inborn metabolic diseases glutaric aciduria type 1 (GA1) and Canavan disease (CD).

### 4.5 Organic anion transport (SLC17 family)

The functionally diverse SLC17 family of organic anion transporters (Reimer, 2013) has been associated with risk for gout, and possibly schizophrenia, as well as amyotrophic lateral sclerosis (ALS), Alzheimer disease, and Huntington disease (VGLUTs).

### 4.6 Mitochondrial transporter (SLC25 family)

In recent years, the completion of sequencing of the human genome and progress in understanding the functional roles of mitochondrial transporters has opened the door to the discovery of a considerable number of diseases associated with defective mitochondrial transporters. These are described in detail in the SLC25 mini-review of Palmieri (Palmieri, 2013) and for the mitochondrial ADP/ATP carrier in the article of Clémençon et al. (Clémençon et al., 2013), and encompass for example ADP/ATP carrier (AAC1) deficiency (exercise intolerance, muscle pain, progressive hypertrophic cardiomyopathy), phosphate carrier (PiC) deficiency (muscular hypotonia, progressive hypertrophic cardiomyopathy), aspartate/glutamate carrier isoform 1 (AGC1) deficiency (severe hypotonia, psychomotor developmental arrest, seizures, spasticity), neuropathy with bilateral striatal necrosis (flaccid paralysis and encephalopathy, bilateral striatal necrosis, chronic progressive polyneuropathy), and congenital sideroblastic anemia (severe anemia with hypochromia, microcytosis and ringed sideroblasts in the bone marrow).

### 4.7 Anion transporters (SLC26 family)

Alper and Sharma (Alper and Sharma, 2013) report, in their review of the SLC26 gene family of anion transporters and channels, mutations in three human SLC26 genes being associated with congenital or early onset Mendelian diseases: chondrodysplasias for *SLC26A2*, chloride diarrhea for *SLC26A3* and deafness for *SLC26A4*. Additional disease phenotypes evident only in mouse knockout models include oxalate urolithiasis, gastric hypochlorhydria, distal renal tubular acidosis, and male infertility.

### 4.8 Zinc transport (SLC30 and SLC39 families)

The SLC30 and SLC39 families encompass genes encoding zinc transporters and are introduced in the reviews of Huang and Tepaamorndech (Huang and Tepaamorndech, 2013) and Jeong and Eide (Jeong and Eide, 2013). A point mutation in the *SLC30A2* gene has been demonstrated to be responsible for an autosomal dominant disease of zinc metabolism in humans (transient neonatal zinc deficiency). Abnormal zinc metabolism has also been shown to be associated with the risk of diabetes, breast cancer and prostate cancer (Franz et al., 2013).

### 4.9 Riboflavin transport (SLC52 family)

The SLC family of riboflavin transporters (RFVT, SLC52) (Yonezawa and Inui, 2013) has been linked to multiple acyl-CoA dehydrogenase deficiency (MADD), an autosomal recessive disorder mainly affecting amino acid and fatty acid metabolism, and Brown-Vialetto-Van Laere Syndrome, a rare autosomal recessive neurologic disorder characterized by sensorineural hearing loss and a variety of cranial nerve palsies.

## 5 Role of transporters in the drug approval process

Due to the known roles of many SLC family members in drug disposition, transporters are now being evaluated as a routine part of the drug development process and are receiving attention by regulatory authorities around the world. The recognition of the roles of transporters in both drug distribution and drug-drug interactions has become a topic around which the US-FDA launched an expert panel review leading to a white-paper from which recommendations and guidelines have now been issued by both the US-FDA and the European Medicines Agency (EMA). In this series, Maeda and Sugiyama (Maeda and Sugiyama, 2013) review the current regulatory and scientific status of transporter evaluation as part of the drug approval process.

## 6 Pharmaceutical perspective

In addition to the roles of transporters in the diseases noted above, several transporters are also of great importance from a pharmaceutical perspective. For example, transporters can serve as drug targets or as a mechanism to facilitate drug delivery to cells and tissues. Recently exploited drug transporter targets include neurotransmitter transporters (SLC6 family), intestinal bile acid transporters (SLC10 family) and cation-Cl cotransporters (SLC12 family). In a very recent announcement, dapaglifloxin (Forxiga), the first of a class of inhibitors of the SGLT2 (SLC5A2) sugar transporters, has been approved in Europe for treatment of Type 2 diabetes. The intestinal oligopeptide transporter PepT1 (SLC15A1) or transporters at the blood–brain barrier (various SLC families) are proving to be important drug delivery systems. Due to the competitive nature of many of these drug discovery programs, it is difficult to fully assess the current activity of pharmaceutical research centered around transporters. However the examples cited above are likely only the “tip of the iceberg” of opportunity.

## 7 Outlook

It is now clear that our understanding of the roles of transporters in disease, drug disposition, and toxicology is evolving quickly, driven by technical advances in assay development, protein structure determination, and evaluation of the roles physiology in both health and disease. Although we now understand many of the gene families, the interplay between the substrates and ligands with which they interact will remain a challenge to understand more fully. Each of the mini-reviews in this series provides, based on the information known today, a prospective look at the direction of research and opportunities. It is clear that the upcoming years will see us rounding out our understanding of all the transporter genes, and their functions and roles in physiology will elucidate exciting and possibly unexpected opportunities for the understanding and treatment of human diseases. As protein structures become ever more available, improved strategies emerge using this information, along with improvements in rational drug discovery approaches, to facilitate development of both new drugs as well as research tools to more deeply understand the biological roles transporters play. The future challenge for the scientific community will be the biochemical, biophysical, physiological and pharmacological assessment of all these novel gene products in a manner that can be used to improve our understanding of transporter biology, with a focus on human physiology, pathophysiology and drug discovery.
